# Mood State Detection in Handwritten Tasks Using PCA–mFCBF and Automated Machine Learning

**DOI:** 10.3390/s22041686

**Published:** 2022-02-21

**Authors:** Juan Arturo Nolazco-Flores, Marcos Faundez-Zanuy, Oliver Alejandro Velázquez-Flores, Carolina Del-Valle-Soto, Gennaro Cordasco, Anna Esposito

**Affiliations:** 1 School of Engineering and Science, Tecnológico de Monterrey, Monterrey 64849, Mexico; a01380501@itesm.mx; 2 Escola Superior Politecnica, TecnoCampus Mataro-Maresme, 08302 Mataro, Spain; faundez@tecnocampus.cat; 3 Facultad de Ingeniería, Universidad Panamericana, Zapopan 45010, Mexico; cvalle@up.edu.mx; 4 Dipartimento di Psicologia, Università della Campania ‘Luigi Vanvitelli’ and IIASS, 81100 Caserta, Italy; gennaro.cordasco@unicampania.it (G.C.); iiass.annaesp@tin.it (A.E.)

**Keywords:** autoML, data augmentation, negative mood states recognition, feature extraction, SVM

## Abstract

In this research, we analyse data obtained from sensors when a user handwrites or draws on a tablet to detect whether the user is in a specific mood state. First, we calculated the features based on the temporal, kinematic, statistical, spectral and cepstral domains for the tablet pressure, the horizontal and vertical pen displacements and the azimuth of the pen’s position. Next, we selected features using a principal component analysis (PCA) pipeline, followed by modified fast correlation–based filtering (mFCBF). PCA was used to calculate the orthogonal transformation of the features, and mFCBF was used to select the best PCA features. The EMOTHAW database was used for depression, anxiety and stress scale (DASS) assessment. The process involved the augmentation of the training data by first augmenting the mood states such that all the data were the same size. Then, 80% of the training data was randomly selected, and a small random Gaussian noise was added to the extracted features. Automated machine learning was employed to train and test more than ten plain and ensembled classifiers. For all three moods, we obtained 100% accuracy results when detecting two possible grades of mood severities using this architecture. The results obtained were superior to the results obtained by using state-of-the-art methods, which enabled us to define the three mood states and provide precise information to the clinical psychologist. The accuracy results obtained when detecting these three possible mood states using this architecture were 82.5%, 72.8% and 74.56% for depression, anxiety and stress, respectively.

## 1. Introduction

Morphological biometrics, based on quantitative measures of the human body [1,2], as well as behavioural biometrics, based on the patterns of actions performed by a subject, have proved to be helpful for e-security and e-health [3]. This research focuses on behavioural biometrics. We analyse the online activities performed during certain specific drawing and handwriting tasks performed by the subjects [4]. For monitoring health conditions, behavioural biometrics, especially online handwriting/drawing, has proved to be more useful in indicating states of mental disorders and diseases, such as dementia, than other popular morphological biometrics traits, such as fingerprint and iris recognition [3,4]. Also, behavioural biometrics is a minimally invasive methodology because it is based on tasks that are part of routine functional activities.

**Figure 1:**
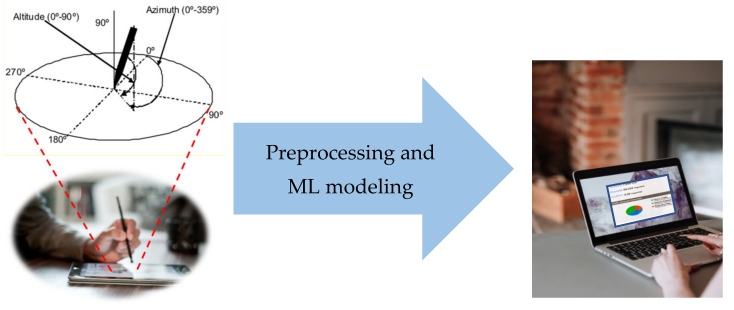
Capture of sensor data from the tablet and pen when handwriting or drawing on a tablet. Sensor data are processed and sent to a clinical psychologist for analysis.

Figure 1 represents the tablet application that captures the sensor data of the tablet and the pen when the user handwrites or draws on the tablet.

Treatment of mental illnesses is a health priority because they significantly impact human well-being and are among the major causes of inabilities in populations worldwide. Indeed, depression, stress and anxiety are the most prevalent negative moods in the world, and stress is often present as a comorbidity. It is estimated that the number of people affected by depression (resp. anxiety) is 4.4% (resp. 3.6%) of the global population [5], and these numbers are rapidly increasing because of the global spread of the coronavirus disease 2019. The manifestation of such disorders is commonly accompanied by the deterioration of social behaviour mainly because of the inability to express one’s emotions and decode others’ moods [6]. Unfortunately, these diseases do not have a definitive treatment, and treatments can last for the entire lifetime with a consistent impact on the quality of life of patients and on the health costs of public administrations. Therefore, it is imperative to detect early indicators of mental disorders to provide timely treatment so that they do not become chronic and difficult to treat. Identifying early indicators hence enables early implementation and effective interventions, which reduces public health care costs [7]. These indicators include changes in a person’s voice, facial expressions and body postures as well as changes in behaviours and functional abilities, such as handwriting and drawing [8].

We focus on negative moods (depression, stress and anxiety) because they last for long periods and a negative state of mind (mood) deteriorates the quality of life of patients. Depression is a mood disorder that causes energy and interest to disappear, and instils a persistent feeling of sadness, which results in high energy consumption by the brain; this can lead to various emotional and physical problems. Clinical depression can be serious; in fact, when depression is left untreated, it can lead to suicide [9].

Anxiety is a cognitive-affective response characterised by feelings of tension and worry regarding a potentially negative outcome that the individual perceives as highly probable and imminent [10]. Anxiety signs and symptoms include nervous sweating, increased heart rate and hyperventilation [9]. It impacts the activities of the patients because this state causes tiredness [11].

Stress is a natural reaction to the pressure that the body undergoes when faced with complicated or dangerous life situations [12]. In general, stress is a normal human response and is part of life, but it becomes a mood disorder when it is experienced frequently and interferes with the ability to perform daily activities. Moreover, when facing stressful situations, the body releases large amounts of several hormones, which can damage the body (causing diabetes and cardiovascular diseases) and cognitive processes [13].

The use of behavioural biometrics, especially, in particular, the online analysis of the activity of a subject performing a handwriting or drawing task enables the characterisation of mood states, especially, depression, anxiety and stress [14]. The use of technological tools (e.g., smartphones, tablets and touch screens) and the multiple interactions among subjects on social media, public administration or health platforms provide access to a large amount of data; this is helpful for discovering or evaluating important features of the subject’s condition.

The characterisation of mood detection through behavioural biometrics, in particular, by the online analysis of handwriting and drawings is a novel and promising research field. Unfortunately, to the best of our knowledge, there are few studies and very few datasets that can be used as a benchmark for potential applications.

This research is based on a study published by Likforman-Sulem et al. [15]; they proposed a methodology to use online handwriting/drawing data to discriminate depressed, stressed and anxious patients from a healthy control group. Their work shows the use of various features to discriminate among negative moods (depression, anxiety and stress) with significant accuracy, sensitivity and specificity by using random forest classification. These features are based on several factors: the duration for which the pen is used on the sheet or near it (in the air), the total time to complete a specific handwriting/drawing task and other features based on the number of strokes performed during a task and/or the pressure applied by the pen on the paper,

In this research, we have improved upon the classification accuracies as compared with our previous research [15] by using principal component analysis (PCA) and modified fast correlation–based filtering (mFCBF) strategies. However, we had to compromise on the explainability of the results. It was not possible to translate the principal components (PCs) into specific sets of kinematic, temporal and pressure variables for any given handwriting task. Clinicians who tried to apply these findings to their clinical settings could not perform a manual handwriting analysis even though we had provided a list of explainable features in Table II of [15]. This can be done easily for an automatic machine system that classifies moods by using online handwritten tasks.

In this research, we test our system using the EMOTHAW database. This database uses the same software and hardware that we had already used when detecting Parkinson’s disease. The main difference is that the EMOTHAW database uses other handwriting/drawing patterns to detect mood states. Therefore, we can use the same features to characterise the user’s data.

In our work on Parkinson’s disease detection [16], we found it useful to add kinematic and statistical features; therefore, the first contribution of this work is to add these features to the user’s features that we used in [17]. The second contribution of this work is the use of a PCA–mFCBF pipeline; in fact, it is probably the most important contribution. In this paradigm, the features are orthogonalised using PCA. Then, the PCs are selected using mFCBF [17]. The third contribution of this work is the use of the automated machine learning (AutoML) approach. We proposed to use AutoML because we had successfully used it in our research on Parkinson’s disease. These three contributions allow for achieving a level of accuracy in the results that highly outperformed state of the art results in mood detection [16].

The last contribution of this research is assessing the detection of three mood states and allowing a high level of precision to the clinical psychologists. This was possible because we achieved 100% accuracy when detecting two mood states.

Section 2 reviews the theory of PCA, which is one of the key concepts used in this work. Section 3 describes the EMOTHAW dataset, which is used in this research to test our methodology. This section also contains information about the distribution of scores for two and three mood states and the explanation of the overlapping of mood states. Section 4 describes the data captured from the tablet and pen. Section 5 describes the temporal, kinematic, statistical, spectral-domain and cepstral-domain features. This section also includes the augmentation method used in this work to synthetically increase the training dataset. Section 6 describes the feature selection (FS) methodology, which includes the PCA [18,19,20,21], mFCBF [17] and the new proposed PCA–mFCBF pipeline. Section 7 describes the hyperparameters of the front end. Section 8 defines the machine learning (ML) modelling to maximise the accuracy detection task. Section 9 reviews the AutoML concepts and AutoML H2O platform used in this work. Section 10 describes the experiments conducted and their results. Finally, in Section 11, we state our remarks and conclusion.

## 2. Principal Component Analysis (PCA)

PCA is useful when multi-colinear vectors are present. PCA can be used to reduce the dimensions and variances of the vectors and to denoise them.

Given the set of possible correlated feature vectors FV, PCA applies an orthogonal transformation to obtain a set of linearly uncorrelated observations: the PCs. This is achieved by projecting the original features into a reduced PCA space using the eigenvectors, which are the PCs of the covariance matrix. The number of principal components obtained by applying PCA is less than or equal to the minimum values between the number of observations $O_{i}$ and the number of features [18,19,20,21]. The resulting projected features are a linear combination of the original features, which capture most of the feature variances. In this transformation, the first component explains the maximum variance in the features, and each subsequent PC explains less of the variance. Most of the useful PCs are dictated by the rank of the matrix.

The variance–covariance matrix is defined as follows:

$${\mathbb{C}}_{X}=\frac{1}{n}X^{T}X$$

X is real symmetric matrix; therefore, the above expression can be decomposed as

$${\mathbb{C}}_{X}=U\Lambda U^{T}$$

where U represents the PCs and is an orthogonal matrix whose columns are eigenvectors of X, and Λ is a diagonal matrix whose entries are the eigenvalues of X [22].

Then, the projected features can be expressed as follows:

Y=XU

The PCA transformation corresponds to multiplying the original features X by the transformation matrix U that represents the PCs. In other words, Y can be viewed as a linear regression, that is, each element in Y can be predicted with a linear combination of the original feature vector X weighted for a vector in the matrix U. ${\mathbb{C}}_{Y}$ is a diagonal matrix that is defined as follows:

$${\mathbb{C}}_{Y}=\frac{1}{n}Y^{T}Y$$

Now, substituting for Y, we obtain

$${\mathbb{C}}_{Y}=\frac{1}{n}{\left(XU\right)}^{T}\left(XU\right)=\frac{1}{n}U^{T}X^{T}XU,\;{\mathbb{C}}_{Y}=U^{T}\left(\frac{1}{n}X^{T}X\right)U=U^{T}{\mathbb{C}}_{X}U.$$

Substituting ${\mathbb{C}}_{X}=U\Lambda U^{T}$, we obtain

$${\mathbb{C}}_{Y}=U^{T}\left(U\Lambda U^{T}\right)U=U^{T}U\Lambda U^{T}U$$

**Figure 2:**
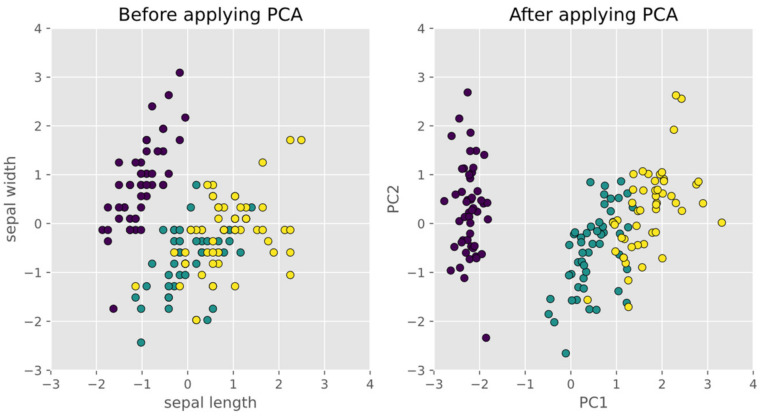
Three classes before and after applying PCA. Three classes before and after applying PCA. In black are shown Iris-setosa’s observations; in green are shown Iris-versicolor’s observations; in yellow are shown Iris-virginica’s observations.

Finally, we obtain

$${\mathbb{C}}_{Y}=\Lambda ,$$

which is a diagonal matrix; this implies that all PCs are uncorrelated with one another. For example, Figure 2 shows the sepal length and sepal width features before and after applying PCA for the well-known Iris dataset [23]. We can see that the first PC variance PC1 is greatly reduced after applying PCA.

## 3. EMOTHAW Databases

In [15], the authors defined a database named EMOTHAW, which they abbreviated from the phrase ‘emotion recognition from handwriting and drawing’; this database has 129 participants whose mood states have been assessed using the depression, anxiety and stress scale (DASS) questionnaire. For each subject, this database includes raw data recorded through a digitising tablet. DASS has approximately seven handwriting/drawing tasks based on a set of well-assessed tests in the medical domain, namely, clock-drawing test, mini-mental state examination test, house–tree–person test and four other simple tasks [24,25].

### 3.1. The DASS Scale

DASS is a 42-item self-report questionnaire developed in [26]. The Italian version (I-DASS-42) was assessed by Severino [27]. DASS consists of three scales that measure the three related negative mood states of depression, anxiety and stress through a 14-point questionnaire. The score ranges are given in Table 1. The DASS scores help establish a bridge between the tasks and moods because the scores indicate whether stress, depression and anxiety were of a normal, mild, moderate, severe or extremely severe degree. The range values and usual interpretations (labels) for the three mood states are shown in Table 1.

The database contained only 129 subjects; therefore, the detailed classification (described in Table 1) would have generated outliers for most of the labels. In [15], the authors adopted a binary classification of mood states for the DASS scores; Table 1 shows the two mood states: normal and above normal. A person who scores higher than 9 on the depression scale is considered to be depressed. A person who scores higher than 7 on the anxiety scale is considered to be anxious. A person who scores higher than 14 on the stress scale is considered to be stressed.

In this paper, we define trinary classification of mood states for the DASS scores; Table 1 shows these three mood states: normal, mild and moderate. A user scoring a value less than 10 on the depression DASS scale is considered to be normal. A person who scores between 10 and 13 is considered to be mildly depressed. A person who scores more than or equal to 14 is considered to be moderately depressed. A user scoring a value of less than 8 on the anxiety DASS scale is considered to be normal; a score of 8 or 9 is considered to be mild depression; and a score higher than or equal to 10 is considered to be above mild depression. A user scoring a value less than 15 in the stress DASS scale is considered to be normal; a score between 15 and 18 is considered to be mild stress; and a score greater than or equal to 19 is considered to be moderate stress.

### 3.2. Subjects

The EMOTHAW database consists of data obtained from 129 participants (71 females and 58 males) aged between 21 and 32 years with a similar demographic background, to ensure controlled experimentation. All the subjects were right-handed and were all students of Università degli Studi della Campania L. Vanvitelli in Italy. The data acquisition protocol consisted of filling in the DASS questionnaire (Italian version), followed by the execution of seven handwriting/drawing tasks described below.

### 3.3. Tasks

The recorded tasks performed by each subject are shown in Table 2.

**Figure 3:**
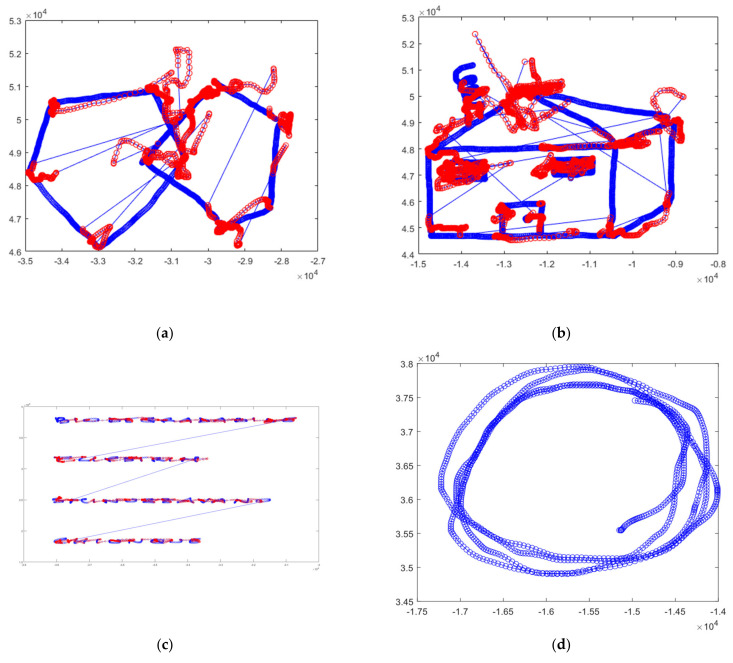

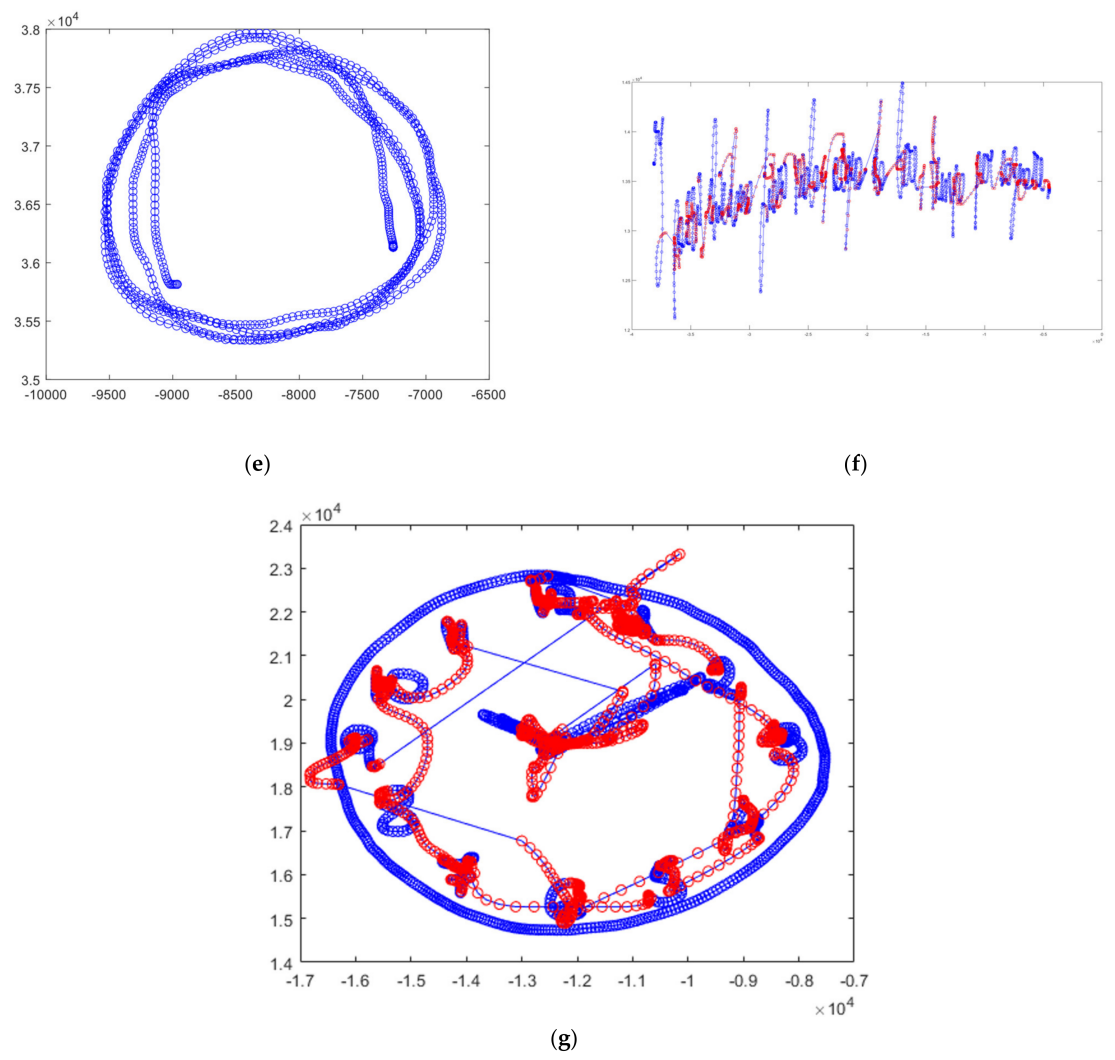
Examples of drawings for different tasks: (**a**) overlapping pentagons, (**b**) a house, (**c**) words, (**d**) circular loops drawn with the left hand, (**e**) circular loops drawn with the right hand, (**f**) a cursive sentence and (**g**) a clock.

Figure 3 shows an example of the different tasks performed by the participants. Figure 3a shows the pentagon-drawing task. Figure 3b shows the house-drawing task. Figure 3c shows an example of the four Italian words in uppercase letters (BIODEGRAD-ABILE (biodegradable), FLIPSTRIM (flipstrim), SMINUZZAVANO (to crumble), CHIUNQUE (anyone) [15]. Figure 3d,e show examples of the left- and right-hand loop drawings, respectively. Hand loop drawings are the simplest tasks but still provide good information. For example, we can observe users’ motor activities in their muscles. Figure 3f shows an example of the cursive writing of a sentence. Figure 3g shows an example of the clock-drawing task.

For data acquisition, the authors of [15] tested the differences between the online and offline data collection methods and concluded that collecting online data had more impact for the research because information, such as the pressure, pen position and time stamp, could be obtained in this way. In their article, they proved the relevance of these characteristics.

The analyses in [15] were based on four signals: time in the air (pen status = 0), time on paper (pen status = 1), the total duration of the task and the number of strokes. Then, the authors selected five tasks and extracted the four abovementioned features for each task; there were a total of 20 features. They compared the contributions of handwriting-based tasks (two tasks = eight features), drawing-based tasks (three tasks = twelve features) and the combination of the two tasks (20 features). The experimentation exploits a random forest model for mood state detection using the leave-percentage-out (LPO) approach that repeats the experiments ten times for each type of task.

In [15], the results showed that the writing-based tasks were less effective than the other tasks in all the considered mood states, particularly on stress. For depression, the drawing-based tasks were the most effective, whereas a combination of drawing and writing tasks was the most effective solution for characterising stress and anxiety.

### 3.4. Distribution of Scores for Two and Three Mood States

**Figure 4:**
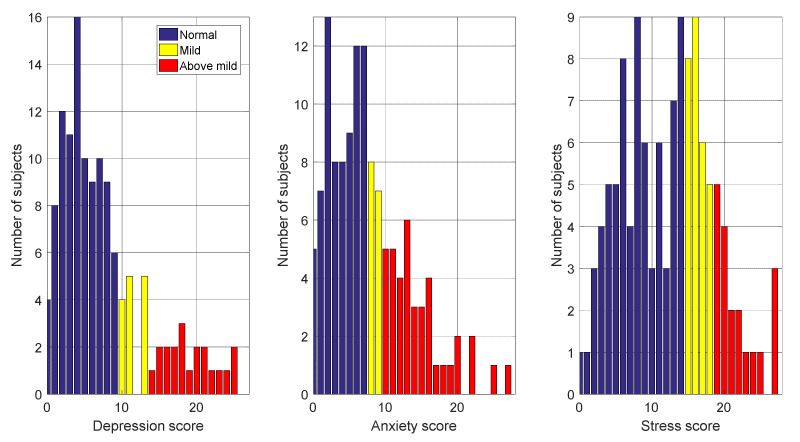
DASS score distribution in the EMOTHAW database. For binary labelling, the dark blue bars show normal scores, and the yellow and red bars show above-normal scores. For trinary labelling, the dark blue bars show normal scores, the yellow bars show mild scores and the red bars show above mild scores.

The distributions of DASS scores in the EMOTHAW database are shown in Figure 4. For binary labelling, the bars in dark blue are the normal scores, and the bars in yellow and red show the above-normal scores. For binary labelling, the proportions of depression, anxiety and stress were approximately 67%, 58% and 57%, respectively. For trinary labelling, the bars in dark blue are the normal scores, the bars in yellow are the mild scores and the bars in red are the moderate scores. Moreover, these classes are highly unbalanced.

### 3.5. Overlapping of Mood States

The consistency and temporal stability of the DASS scales have been assessed in several studies [15]. In this study, participants performed the writing or drawing tasks as soon as they completed the DASS questionnaire. Therefore, we are confident that the participants performed the tasks under the measured states.

**Figure 5:**
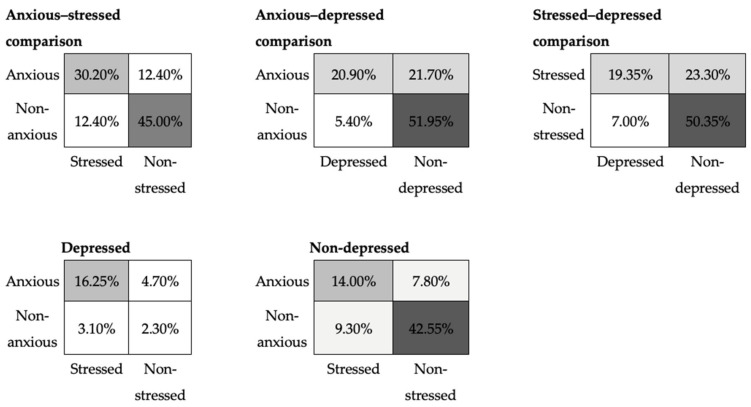
Cross-tables showing the percentage of co-occurrence of mood states in the EMOTHAW database.

The cross-tables in Figure 5 show that depression, anxiety and stress can be observed separately or simultaneously. The scores have been dichotomised. From the matrices in the second row of Figure 5, we observe that for approximately 20% of the participants (19.4% = 2.3% + 7.8% + 9.3%), a single negative state is observed (such as anxious/non-stressed/non-depressed). For approximately the same percentage of participants (21.8% = 3.1% + 4.7% + 14.0%), two negative mood states were observed simultaneously. However, due to the construction of the scales, each of these mood states can be predicted separately. A Pearson’s *χ*2 test conducted on the anxious/stressed, stressed/depressed and anxious/depressed cross tables showed that the qualitative variables anxiety–stress, stress–depression and depression–anxiety were linked (*p*-values below 0.01). The strongest link is between anxiety and stress. The next strongest link is between anxiety and depression. The weakest of the three links is that between stress and depression.

We did not analyse the comorbid mood states because the number of available samples in EMOTHAW for certain combinations was too small.

## 4. Sensors Data

**Figure 6:**
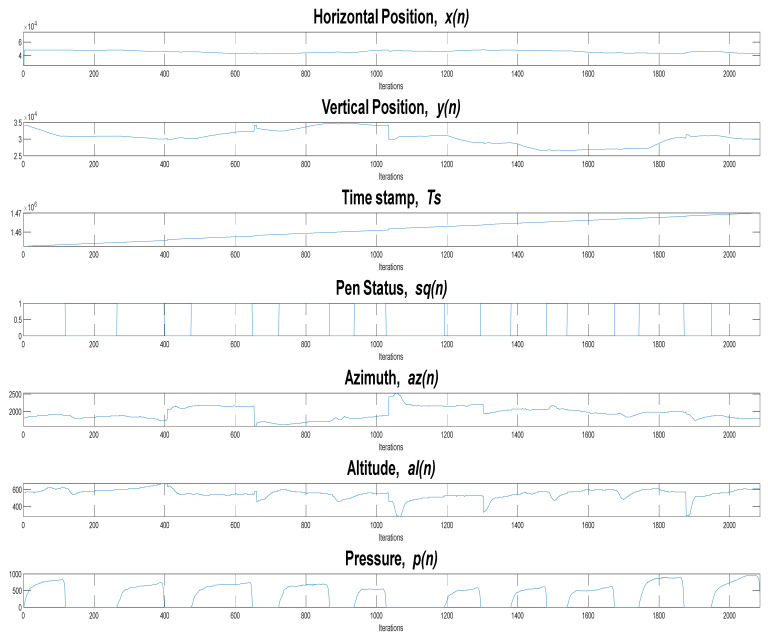
Online time-series drawing for the pentagon-drawing task.

Figure 6 is a graphical representation of the signals captured in real time by the software when drawing on the tablet. These signals are as follows:

- Horizontal position or displacement of the pen tip along the x-axis, $x\left(n\right)$;
- Vertical position or displacement of the pen tip along the y-axis, $y\left(n\right)$;
- Timestamps (in milliseconds), $t\left(n\right)$;
- Pen status, that is, on-surface/in-air pen position status (touch/no-touch the paper), $sq\left(n\right);$
- Altitude angle of the pen with respect to the tablet’s surface, $al\left(n\right)$;
- Azimuth angle of the pen with respect to the tablet’s surface, $az\left(n\right)$;
- Pressure applied by the pen tip on the tablet’s surface, $p\left(n\right)$.

All tasks have a duration of T seconds (N samples).

### Data Augmentation

In the EMOTHAW database, the classes for binary and trinary labelling are highly unbalanced. Therefore, to improve accuracy, the training data needed to be augmented. First, we augmented the data such that all the mood states had the same number of observations. Then, we augmented all the mood states using the following steps:

1. Identify the mood states having few observations,
2. Calculate the number of samples required to make all the mood state observations of the same size,
3. Randomly select observations from the original data and
4. For each selected sample, calculate the new feature vector by adding the Gaussian random noise to the original features:

$$FV_{a}^{u*}=FV^{u*}+\alpha *GV,$$

where $FV^{u*}$ is the feature vector of a random user, and α is a scale value lower than 0.2. GV is a vector with the same dimension as $FV^{u*}$ with the Gaussian random values having mean and variance values of 0 and 1, respectively.

## 5. Feature Extraction

**Figure 7:**
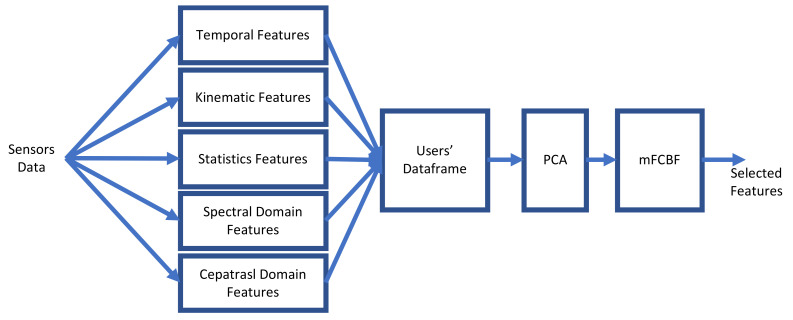
System front-end that starts with the temporal features, kinematic features, statistical features, spectral-domain features and cepstral-domain features. These features are concatenated; then, they are orthogonalised using PCA. Finally, the features are selected using our mFCBF.

The system front-end used in this research is shown in Figure 7. There are two main differences between this research and our previous study on mood state detection [16]. The first difference is the addition of kinematic and statistical features in this study. We added these features because they proved to be effective in increasing the accuracy of the results obtained in our study on Parkinson’s disease detection [17]. The second and most important difference is the inclusion of PCA, which is used to orthogonalise all features. The inclusion of PCA as orthogonalisation before selection is novel because, instead of selecting features by using the first PCA’s coefficients, we selected features by applying mFCBF on the PC. In other words, we pipelined PCA and mFCBF.

### 5.1. User’s Features

In this paper, we will use the following set of feature vectors for different feature sets:

$$FV_{T}^{u}=TF^{u}\;FV_{T\_SD\_CD}^{u}={\left[{\left[TF^{u}\right]}^{T}\cup {\left[SDF^{u}\right]}^{T}\cup {\left[CF^{u}\right]}^{T}\right]}^{Τ}\;FV_{T\_K\_S\_SD\_CD}^{u}={\left[{\left[FV_{T\_SD\_CD}^{u}\right]}^{T}\cup {\left[KF^{u}\right]}^{T}\cup {\left[SF^{u}\right]}^{T}\right]}^{T}={\left[{\left[TF^{u}\right]}^{T}\cup {\left[KF^{u}\right]}^{T}\cup {\left[SF^{u}\right]}^{T}\cup {\left[SDF^{u}\right]}^{T}\cup {\left[CF^{u}\right]}^{T}\right]}^{Τ},$$

where $FV_{T}^{u}$ is the feature vector for the temporal features [16]; $FV_{T\_SD\_CD}^{u}$ is the feature vector of the temporal, spectral- and cepstral-domain features; $FV_{a}^{u}$ is the feature vector obtained by concatenating the kinematic and statistical features to $FV_{T\_SD\_CD}^{u}$; $TF^{u}$ is the raw vector of the temporal features of the user u as defined in Table 3; $KF^{u}$ is the raw vector of the kinematic features of the user u as defined in Table 4; $SF^{u}$ is the raw vector of the statistical features of the user u, as defined in Table 5; $SDF^{u}$ is the raw vector of the log energy filterbank features of the user u in the spectral domain; and $CF^{u}$ is the raw vector of the cepstral features calculated as the Fourier transform of the log energy of the filterbanks of the user u. All these features are calculated for all the tasks, as follows:

$$T=\left\{spiral,letter\;l,\;\mathrm{syllable}\;le,\;trigramm\;les,word1,\;word2,\;word3,\;sentence\right\}$$

### 5.2. Detection Task

The detection task involves identifying the mood state of the user. For binary labelling, we define the mood state for each user as follows:

$$S^{u}=\left\{\begin{array}{c} 0,\;Normal\\ 1,\;above\;Normal \end{array}\right.\;for\;all\;u=1\ldots U,$$

Therefore, we can relate the feature vector F with the corresponding mood state as follows:

$$FVS_{F}^{u}={\left[{\left[FV_{F}^{u}\right]}^{Τ}\cup {\left[S^{u}\right]}^{Τ}\right]}^{Τ},$$

where $F=\left\{T,\;T\_SD\_CD,\;T\_K\_S\_SD\_CD\right\}$. A dataframe is defined as the union of all users U in $FVS_{F}^{u}$. Hence, for each feature F, this operation can be expressed using relational algebraic notation as follows:

$$FVS_{F}=\cup_{u=1}^{U}FVS_{F}^{u}.$$

In these data frames, the rows represent the number of users, and the columns represent the features and the disease state of the users.

For trinary labelling, we define the mood state for each user as follows:

$$S^{u}=\left\{\begin{array}{cc} 0 & Normal\\ 1 & Mild\\ 2 & above\;Mild \end{array}\right.\;for\;all\;u=1\ldots U,$$

### 5.3. Features for Moods

The EMOTHAW database includes three moods; therefore, the feature vector for each mood is defined as follows:

$$FVS_{F}^{M},$$

where $M=\left\{Depression,\;Anxiety,\;Stress\right\}$. In our detection task, we detected only the state of one mood at a time.

## 6. Feature Selection

It is important to select the right features because data models also learn irrelevant information, which degrades their performance. There are multiple methods to select features. In this research, we test the accuracy performance measures of PCA, mFCBF and the PCA–mFCBF pipeline.

### 6.1. Principal Component Analysis (PCA)

PCA is an orthogonal transformation of the features; it rotates the dimensional axis to maximise variability. PCA returns coefficients in order of importance—the first coefficient has the highest variance representation in the entire set. The higher the selected PC, the higher the representability of the variance of the set of features.

Let us represent the PCA function on the feature vector F and mood M as follows:

$$PC_{F}^{M}=PCA\left(FVS_{F}^{M}\right)$$

The maximum number of PCs calculated by PCA is equal to the number of observations (in this case, it is equal to the number of users). The selected PCs P of the entire features F is represented as follows:

$${\tilde{FVS}}_{F}^{M}={\left\{PC_{F}^{M}\right\}}_{1}^{P}.$$

As stated earlier, although PCA helps minimise features to improve model accuracy, it has a negative effect on the model’s explainability, which is a drawback.

### 6.2. Modified Fast Correlation-Based Filtering (mFCBF)

The system front-end calculates many features from a limited number of time signals captured from the tablet’s sensor. Beside this correlation, certain features contribute more towards creating an accurate model; mFCBF selects the features that, even when they are correlated, mostly contribute to the increase in model accuracy.

FCBF selection is based on two steps [16]. In the first step, the selected features are those whose correlation with the output are higher than the correlation with the threshold value. The second step takes the features of the first step and selects the features with a correlation less than the threshold value. Algorithm 1 shows the pseudocode of our modified version of the function mFCBF [17]. This modified version differs from the original version in step 5, where the selected feature has high correlation with the output. The mFCBF algorithm receives a data frame and the thresholds oTh and iTh as inputs. Here, oTh is used to set the lower correlation threshold of each of the selected features and the output; the practical value of this parameter should be greater than 0.2. Also, iTh is used to set the higher correlation value between the features; the practical value of this parameter should be less than 0.2. By sweeping oTh and iTh for a range of values, we can find the right features that maximise the performance of the ML method. This operation for the features F and mood M is expressed as follows:

$${\hat{FVS}}_{F}^{M}\left(oTh,iTh\right)=mFCBF_{oTh,iTh}\left(FVS_{F}^{M}\right)$$

for 0≤iTh≤0.2 and 0.2≤oTh≤1.0.

Note that these equations represent 2D arrays, where one dimension represents the number of users, and the other dimension represents the number of selected features.

**Algorithm 1.** The mFCBF algorithm receives the users’ feature matrix (O), minimum correlation threshold (oTh) and the maximum correlation threshold (iTh) and returns the selected set of features.1: Function mFCBF (FVS, oTh, iTh) 2: Calculate corr (O) 3: $FVS_{tmp}\;\leftarrow$ Select columns whose correlation with the output is > oTh 4: Calculate corr ($FVS_{tmp}$) 5: $FVS_{oTh,\;iTh}\leftarrow$ Select columns whose correlation with the input is < iTh and with the highest correlation with the output. 6: Return ($FVS_{oTh,\;iTh}$) 7: End function

### 6.3. PCA-mFCBF Pipeline

In Section 6.1 and Section 6.2, we selected features using PCA or mFCBF, respectively. In this section, we propose to pipeline them by first applying PCA and then applying mFCBF. The PCA step returns all the PCA coefficients. Then, the selection step is performed using mFCBF. The intra-feature variability is already minimised; therefore, this step selects the PC which has a higher correlation with the output.

This PCA–mFCBF pipeline for the features F and mood M is defined as follows:

$${\overset{=}{FVS}}_{F}^{M}\left(oTh,iTh\right)=mFCBF_{oTh,iTh}\left(PC_{F}^{M}\right).$$

In Section 10, we will prove that orthogonalising before selection with mFCBF is a good strategy to greatly increase the accuracy.

Again, the purpose of mFCBF in this pipeline is to select the PCs’ that contribute the most to increasing the model accuracy. Although it is beneficial to orthogonalise features using PCA to improve the model’s accuracy, a potential drawback of PCA is its adverse effect on the model’s explainability.

## 7. Front-End Hyperparameters

Spectral-domain features $\left(SDF\right)$ and cepstral-domain features (CDF) are functions of parameters, such as filterbank bandwidth $\left(fbbw\right)$, bandwidth of the filters in the filterbank $\left(fbw\right),$ filterbank initial frequency $\left(if\right)$ and overlap of the filters in the filterbank $\left(ov\right)$. However, FS depends on the feature-output-correlation threshold (oTh) and the intra-feature correlation threshold (iTh). All these parameters depend on the next set of parameters: fbbw, fbw, if, ov,oTh,iTh. Therefore, we must tune these parameters to optimise the model’s accuracy.

The range of values for each of the parameters are defined as follows: $iTh_{range}=\left[0.2-1\right]$, and $oTh_{range}=\left[0-0.20\right]$. For similar signals on the Parkinson’s disease database [17], we set $fbbw_{range}$ = 30 Hz $fbw_{range}=1$ Hz, $if_{range}=0.5$ Hz and $ov_{range}=0\%$. The discrete versions of $iTh_{range}$ and $oTh_{range}$ are $iTh_{range}^{s}=\left[0.2,\;0.30,\;\ldots ,1.0\right]$ and $oTh_{range}^{s}=\left[0,0.02,0.04,0.06,\ldots ,0.18,0.20\right]$.

## 8. ML Modelling to Maximise the Detection Task’s Accuracy

Let us define $ML_{F}^{M,MLm}$ as the ML model for mood $M=\left\{Depression,\;Anxiety,Stress\right\}$, ML model in $MLm=\left\{SVM,autoML\right\}$, and feature in $F=\left\{T,\;T\_SD\_CD,\;T\_K\_S\_SD\_CD\right\}$. Therefore, the optimisation of the ML model that maximises accuracy for the ML method MLm, features F and mood M is defined as follows:

$$ML_{F}^{M,MLm}=\underset{iTh_{range}^{s},oTh_{range}^{s}\;}{\max}MLm\left(FVS_{F}^{M}\right)$$

When PCA is used as the FS method, this optimisation can be represented as follows:

$${\tilde{ML}}_{F}^{M,MLm}=\underset{iTh_{range}^{s},oTh_{range}^{s}\;}{\max}MLm\left({\tilde{FVS}}_{T}^{M}\left(oTh,iTh\right)\right).$$

When mFCBF is used as the FS method, this optimisation can be represented as follows:

$${\hat{ML}}_{F}^{M,MLm}=\underset{iTh_{range}^{s},oTh_{range}^{s}\;}{\max}MLm\left({\hat{FVS}}_{F}^{M}\left(oTh,iTh\right)\right).$$

When the PCA–mFCBF pipeline is used as an FS method, this optimisation can be represented as follows:

$${\overset{=}{ML}}_{F}^{M,MLm}=\underset{iTh_{range}^{s},oTh_{range}^{s}\;}{\max}MLm\left({\overset{=}{FVS}}_{F}^{M}\left(oTh,iTh\right)\right)$$

where $M=\left\{Depression,\;Anxiety,\;Stress\right\}$ is the mood; $MLm=\left\{SVM,autoML\right\}$ is the ML model; and $F=\left\{\phi ,T,T\_SD\_CD,\;T\_K\_S\_SD\_CD\right\}$ is the feature set used. The empty set notation ϕ is used when no FS method is used.

For the randomness of the augmentation method, we trained and tested the model for different user sets and random sequences, and we selected the maximum accuracy.

## 9. AutoML

AutoML, also known as augmented ML, is a methodology that aims to automate the data science pipeline for classification and regression. The AutoML pipeline includes data pre-processing (cleaning, imputing and quality checking), feature engineering (transformation and selection), model selection, evaluation and hyper-parameter optimisation.

There are different platforms for implementing AutoML; each platform has a different maturation and state of evolution for continuous improvement. Recently, certain AutoML systems have started to support more focused tasks, such as time-series forecasting. A few of the well-known automated machine platforms are H2O [28,29], PyCaret [30,31], auto-sklearn [32,33], the tree-based pipeline optimisation tool (TPOT) [34,35,36] and MLBox [37].

H2O includes automatic training and tuning of many models within a user-specified time limit. Stacked ensembles are automatically trained on collections of individual models to produce highly predictive ensemble models.

PyCaret is an end-to-end ML and model management tool that speeds up the experiment cycle exponentially and increases productivity.

The auto-sklearn architecture is an AutoML toolkit and a drop-in replacement for the scikit-learn estimator; it includes algorithm selection and hyper-parameter tuning. It uses Bayesian optimisation, meta-learning and ensemble construction.

TPOT uses genetic programming to determine the best performing ML pipelines, and it is built on top of scikit-learn [38]. It supports feature pre-processing, feature construction and selection, model selection and hyper-parameter optimisation.

MLBox supports fast reading, distributed data processing, FS, leak detection, cleaning, formatting, accurate hyper-parameter optimisation in high-dimensional space and ML algorithms, such as deep learning, stacking, light gradient boosting machine and XGBoost. An important feature is that it includes prediction with the interpretation of models.

AutoML packages are not perfect, and there are packages with different levels of automation. For example, feature engineering is a task in this pipeline for which AutoML has some features, but a lot more work is required to develop and automate this task, especially for sensor data analysis.

### AutoML H2O

For data modelling, we used AutoML H2O [28,29], which is a platform that can be used for automating the ML workflow. The automation includes the training and tuning of many models within a user-specified time limit. It also includes hyper-parameter optimisation, which uses a Bayesian approach. Table 6 shows a few of these models, such as variations of trees, random forests, Naïve Bayes, linear models, additive models, deep learning and support vector machines (SVMs). In addition, H2O also includes a model that is an ensemble of all models and is a model for each family of methods. The ensembled models are mostly the ones with better accuracy results.

## 10. Experiments and Results

H2O [28,29] is an AutoML package intended to automate the ML pipeline starting from data manipulation to ML parameter optimisation. Table 7 shows the configuration setting that we used for H2O. In this configuration, we limited the maximum expected training time to 200 s to avoid a long processing time. The number of models was set to 15 as a trade-off between the accuracy performance and processing time. We excluded the gradient boosting machine (GBM) modelling because its database did not converge with our database. The number of folds was set to two because when this was increased, the accuracy of the results did not improve, but the process time did. Finally, the stop metric was set to log loss because this was a classification task.

LPO was used for testing. PLO is a variation of leave-one-out; however, instead of leaving one element out, the data model in LPO was tested with a percentage of the registers in the database, and we trained with the rest. In our experiments, we repeated this training–testing cycle until we circulated all the possibilities, and we averaged the accuracy values of all tests. In our experiments, we always left out 10%.

Augmentation was controlled by the percentage of augmentation and the amplitude of the Gaussian random noise applied to the original signal. In this research, we first augmented the data such that all the mood states had the same number of observations. Then, we augmented all the mood states by 80% using the Gaussian noise with 0 and 1 as the mean and variance values, respectively. The Gaussian random noise amplitude was multiplied by 0.2.

Table 8 shows the accuracy results for the binary detection moods for the temporal feature F=TF, for the ML models $MLm=\left\{SVM,\;aML\right\}$ and the moods $M=\left\{Depression,\;Anxiety,Stress\right\}$. In this table, we can observe that the accuracy results with AutoML are much higher than the results obtained using SVM [16]. This can be explained because AutoML simultaneously evaluates more than 15 classification algorithms.

Table 9 shows the accuracy results for the features F=T_SD_CD for the ML models $MLm=\left\{SVM,\;aML\right\}$ and for the moods $M=\left\{Depression,\;Anxiety,Stress\right\}$. The second and third columns show the accuracy results obtained when PCA is used as the FS method. We can see that accuracy results with AutoML are much higher than the results obtained with SVM.

The fourth and fifth columns of Table 9 show the accuracy results obtained when mFCBF is used as the FS method. Clearly, the accuracy results with AutoML are much higher than the results obtained with SVM. Also, the accuracy results obtained when using mFCBF as the FS method (in the fourth and fifth columns) are much higher than the accuracy results obtained with PCA (in the second and third columns).

Finally, sixth and seventh columns of Table 9 show the accuracy results when the PCA–mFCBF pipeline is applied as the FS method. The accuracy results with AutoML are much higher than the results obtained with SVM. Also, the accuracy results when using the PCA–mFCBF pipeline as the FS method (in the sixth and seventh columns) are much higher than the accuracy results obtained using PCA (in the second and third columns) or mFCBF (in the fourth and fifth columns).

**Figure 8:**
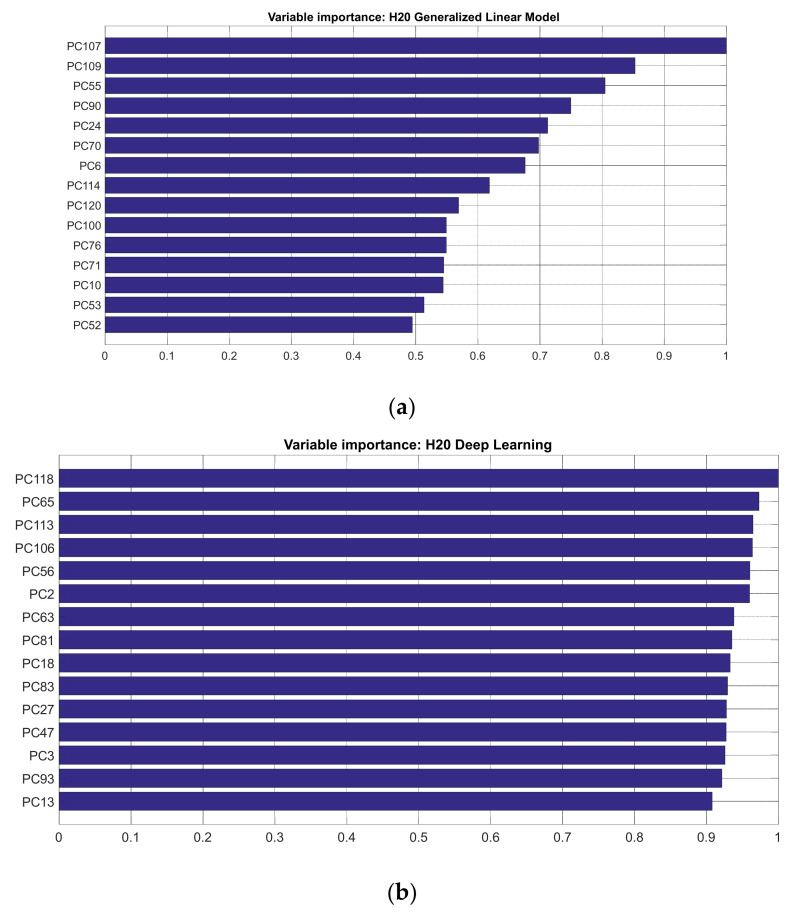

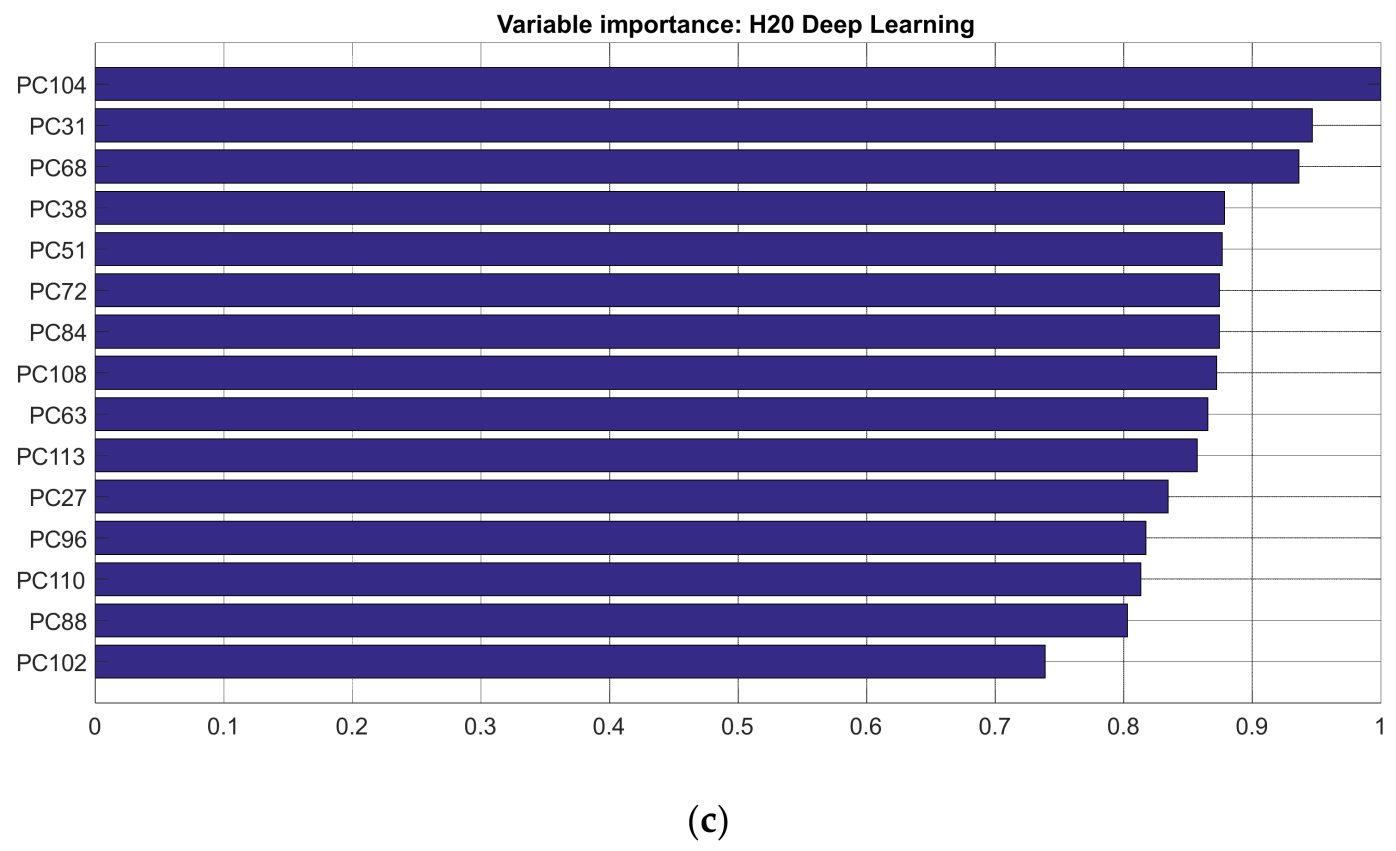
Selected features for (**a**) stress, (**b**) anxiety and (**c**) depression.

Figure 8 shows the selected feature for each task after applying the PCA–mFCBF pipeline. These features are a mixture of the original PCs and are not necessarily the first ones, which are normally selected when PCA is used for FS. This mixture of PCs is because the first step in mFCBF selects the PCs which have a high correlation with the output. Then, the PCs with low correlation are selected.

Table 10 shows the accuracy for the features $F=\left\{T\_SD\_CD,\;T\_K\_S\_SD\_CD\right\}$ for the ML model MLa=autoML and for the moods $M=\left\{Depression,\;Anxiety,Stress\right\}$. The second and third columns are the accuracy results when PCA is used as the FS method. The accuracy results with AutoML, when concatenating the kinematic and statistical features, improved 1.75%, 4.38% and 7.9%, for depression, anxiety and stress, respectively. We can see that the accuracy results are higher when the kinematic and statistical features are concatenated.

The fourth and fifth columns of Table 10 show the accuracy results when mFCBF is used as the FS method. The accuracy results with AutoML, when concatenating the kinematic and statistical features, improved 4.38%, 7.02% and 14.04% for depression, anxiety and stress, respectively. Again, the accuracy of the results is higher when the kinematic and statistical features are concatenated. The accuracy of the results when using mFCBF as the FS method (in the fourth and fifth columns) is much higher than the accuracy of the results that are obtained with PCA (in the second and third columns).

The sixth and seventh columns of Table 10 show the accuracy of the results when the PCA–mFCBF pipeline is used as the FS method. The accuracy results with AutoML, when concatenating the kinematic and statistical features, improved 3.5%, 7.89% and 11.41% for depression, anxiety and stress, respectively.

Clearly, the accuracy of the results when using the PCA–mFCBF pipeline as the FS method (in the sixth and seventh columns) is much higher than the accuracy of the results obtained when using PCA (in the second and third columns) or mFCBF (in the fourth and fifth columns).

Table 11 shows the accuracy of the results for the trinary classification for the features F=T_K_S_SD_CD for the ML model MLa=autoML and for the moods $M=\left\{Depression,\;Anxiety,Stress\right\}$. The second columns is the accuracy of the results when PCA is used as the FS method. The accuracy of the results is higher for the PCA–mFCBF pipeline; the second-best accuracy is obtained with mFCBF; and the third-best accuracy is obtained when using PCA. The accuracy is the worst when no selection feature is used. Therefore, the behavioural biometrics with the trinary classification is identical to the behavioural biometrics with the binary classification.

## 11. Conclusions

In this study, we propose the merging of the temporal, kinematic, statistical, spectral- and cepstral-domain features to detect the mood state. We found that adding the kinematic and statistical features improved the results.

We also proposed to use PCA–mFCBF for FS. PCA is used for orthogonalising features before applying mFCBF to select the features. When using the PCA–mFCBF pipeline, we found that the experimental results were substantially superior to the results obtained when only PCA or mFCBF was used.

The best performance was obtained when adding the kinematic and statistical features pipelined with PCA–mFCBF. The second-best performance was that of the AutoML H2O platform for data modelling. This makes sense because AutoML H2O [28,29] includes the Bayesian hyper-parameter optimisation and model assessment.

The experiment results proved that this pipeline strategy for FS and PCA–mFCBF substantially increases the accuracy results and even reaches 100% in the binary classification of our task.

We also classified data into three categories and developed a few experiments using all the described features. Then, we used PCA–FCBF as the FS method and modelled using the AutoML H2O platform. The accuracy results for trinary detection were 82.45%, 72.8% and 74.56% for depression, anxiety and stress, respectively. Also, we found that the results for the trinary detection were not as impressive as the results obtained for binary detection.

## Figures and Tables

**Table 1:** Interpretation of DASS scores [26]: binary labelling [15] and trinary labelling [this paper].

Binary Labeling Used in [15]	Trinary Labeling	Interpretation of DASS	Depression	Anxiety	Stress
Normal	Normal	Normal	0–9	0–7	0–14
Above normal	Mild	Mild	10–13	8–9	15–18
	Above mild	Moderate	14–20	10–14	19–25
		Severe	21–27	15–19	26–33
		Extremely severe	28+	20+	34+

**Table 2:** Task performed for each user.

Tasks
(1) Drawing a copy of two overlapping pentagons
(2) Drawing a copy of a house
(3) Handwriting of four Italian words in capital letters
(4) Drawing circular loops with the left hand
(5) Drawing circular loops with the right hand
(6) Handwriting of an Italian sentence in cursive letters
(7) Drawing of a clock with twelve hours and hands

**Table 3:** Temporal features for the user u (${TF}^{u}$) for all tasks T.

Notation	Definition
$d^{\tau ,u}\left(n\right)$	Pen’s displacement at the sample n
$S_{1}^{\tau ,u}$	Trajectory taken during handwriting divided by the duration of writing
$F_{1}^{\tau ,u}$	On-air pen duration
$F_{2}^{\tau ,u}$	On-paper pen duration
$d\left(i\right)$	Duration of the stroke i
${\dot{F}}_{2}^{\tau ,u}$	$F_{2}^{\tau ,u}$ normalised to writing duration
$r^{\tau ,u}$	Ratio of time the pen spent in air or on the tablet’s surface
$NCV^{\tau ,u}$	Number of changes in the direction of the velocity vector
$NCA^{\tau ,u}$	Number of changes in direction of the acceleration vector
$NCV_{r}^{\tau ,u}$	$NCV^{\tau ,u}$ relative to writing duration
$NCA_{r}^{\tau ,u}$	$NCA^{\tau ,u}$ relative to writing duration

**Table 4:** Kinematic features for the user u (${KF}^{u}$) for all tasks T.

**Notation**	Definition
$s^{\tau ,u}\left(n\right)$	Stroke signal
$w^{\tau ,u}\left(n\right)$	$\mathrm{Set}\;\mathrm{of}\;\mathrm{discrete},\;\mathrm{horizontal}\;\mathrm{and}\;\mathrm{vertical}\;\mathrm{displacements}\;\left[d^{\tau ,u}\left(n\right),x^{\tau ,u}\left(n\right),y^{\tau ,u}\left(n\right)\right]$
$k_{w}^{\tau ,u}\left(n\right)$	$\mathrm{Set}\;\mathrm{of}\;\mathrm{kinematic}\;\mathrm{signals}\;(v_{w}^{\tau ,u}\left(n\right),a_{w}^{\tau ,u}\left(n\right),j_{w}^{\tau ,u}\left(n\right)),\;\mathrm{of}\;\mathrm{signal}\;\mathrm{in}\;w^{\tau ,u}\left(n\right)$
$v_{w}^{\tau ,u}\left(n\right)$	$\mathrm{Velocity}\;\mathrm{of}\;\mathrm{signals}\;\mathrm{in}\;w^{\tau ,u}\left(n\right)$
$a_{w}^{\tau ,u}\left(n\right)$	$\mathrm{Acceleration}\;\mathrm{of}\;\mathrm{signals}\;\mathrm{in}\;w^{\tau ,u}\left(n\right)$
$j_{w}^{\tau ,u}\left(n\right)$	$\mathrm{Jerk}\;\mathrm{of}\;\mathrm{signal}\;\mathrm{in}\;w^{\tau ,u}\left(n\right)$

**Table 5:** Statistical features for the user u (${SF}^{u}$) for all tasks T.

**Notation**		Definition
$B_{g\left(n\right)}^{\tau ,u}$ (basic statistics features)	${\overset{↔}{g}}^{\tau ,u}$	$\mathrm{Range}\;\mathrm{of}\;\mathrm{signals}\;\mathrm{in}\;g^{\tau ,u}\left(n\right)$
	${\overset{˘}{g}}^{\tau ,u}$	$\mathrm{Median}\;\mathrm{of}\;\mathrm{signals}\;\mathrm{in}\;g^{\tau ,u}\left(n\right)$
	${\ddot{g}}^{\tau ,u}$	$\mathrm{Mode}\;\mathrm{of}\;\mathrm{signals}\;\mathrm{in}\;g^{\tau ,u}\left(n\right)$
	${\dddot{g}}^{\tau ,u}$	$\mathrm{Standard}\;\mathrm{deviation}\;\mathrm{of}\;\mathrm{signals}\;\mathrm{in}\;g^{\tau ,u}\left(n\right)$
	${\overset{\ldots }{\overset{↔}{g}}}^{\tau ,u}$	$\mathrm{Outlier}\;\mathrm{robust}\;\mathrm{range}\;(99\mathrm{th}\;\mathrm{percentile}\;–1\mathrm{st}\;\mathrm{percentile}),\;\mathrm{applied}\;\mathrm{to}\;\mathrm{all}\;\mathrm{signals}\;\mathrm{in}\;g^{\tau ,u}\left(n\right)$
$M_{g\left(n\right)}^{\tau ,u}$ (mean features)	${\overset{↔}{g}}^{\tau ,u}$	$\mathrm{Range}\;\mathrm{of}\;\mathrm{signals}\;\mathrm{in}\;g^{\tau ,u}\left(n\right)$
	${\bar{g}}^{\tau ,u}$	$\mathrm{Arithmetic}\;\mathrm{mean}\;\mathrm{of}\;\mathrm{signals}\;\mathrm{in}\;g^{\tau ,u}\left(n\right)$
	${\overset{=}{g}}^{\tau ,u}$	$\mathrm{Geometric}\;\mathrm{mean}\;\mathrm{of}\;\mathrm{signals}\;\mathrm{in}\;g^{\tau ,u}\left(n\right)$
	$\overset{tri}{\overset{⏞}{g^{\tau ,u}}}$	$\mathrm{Set}\;\mathrm{of}\;\mathrm{trimmed}\;\mathrm{means};\;\mathrm{the}\;\mathrm{mean}\;\mathrm{after}\;\mathrm{removing}\;\mathrm{the}\;\mathrm{outliers}\;\mathrm{for}\;\mathrm{each}\;\mathrm{of}\;\mathrm{the}\;\mathrm{values}\;\mathrm{in}\;\left[5,10,20,30,40,50\right]\;\mathrm{of}\;\mathrm{the}\;\mathrm{signals}\;\mathrm{in}\;g^{\tau ,u}\left(n\right)$
$M_{g\left(n\right)}^{\tau ,u}$ (momentum features)	$\overset{qua}{\overset{⏞}{g^{C}}}$	$\mathrm{Row}\;\mathrm{vector}\;\mathrm{of}\;\mathrm{quartiles}\;(Q_{3}=25(lower,\;Q_{1}=75/upper)\;\mathrm{of}\;\mathrm{signals}\;\mathrm{in}\;g^{\tau ,u}\left(n\right)$
	$\overset{per}{\overset{⏞}{g^{\tau ,u}}}$	$\mathrm{Row}\;\mathrm{vector}\;\mathrm{of}\;\mathrm{percentiles}\;[,5,10,20,30,90,95,100]\;\mathrm{of}\;\mathrm{signals}\;\mathrm{in}\;g^{\tau ,u}\left(n\right)$
	$\overset{mom}{\overset{⏞}{g^{\tau ,u}}}\;$	$\mathrm{Row}\;\mathrm{vector}\;\mathrm{of}\;\mathrm{moments}\;\left[3th,4th,5th,6th\right]$
	$k^{\tau ,u}\;$	$\mathrm{Kurtosis}\;\mathrm{of}\;\mathrm{signals}\;\mathrm{in}\;g^{\tau ,u}\left(n\right)$

**Table 6:** List of Machine Learning Models.

Classification Algorithms
Deep neural network (DNN)
Distributed random forest (DRF)
Extremely randomised trees (ERT)
Generalised linear model (GLM)
Gradient boosting machine (GBM)
Naïve Bayes classifier (NBC)
Rulefit (RF)
Stacked ensembles (SE)
XGBoost (XGB)
Support vector machine (SVM)

**Table 7:** Parameters used when running H2O.

Parameter	Value
max_runtime_secs	200
max_models	15
exclude_algos	GBM
seed	1
nfolds	2
stopping_metric	logloss

**Table 8:** Binary accuracy results for temporal features TF using SVM [16] and AutoML.

M	$ML_{T}^{M,SVM}$ **[16]**	$ML_{T}^{M,aML}$
Depression	71.47	80.70
Anxiety	58.53	71.93
Stress	61.24	66.67

**Table 9:** Binary accuracy results for PCA, mFCBF and PCA-mFCBF feature selection methods using SVM and autoML.

M	${\tilde{ML}}_{T\_SD\_CD}^{M,SVM}$	${\tilde{ML}}_{T\_SD\_CD}^{M,aML}$	${\hat{ML}}_{T\_SD\_CD}^{M,SVM}$ **[16]**	${\hat{ML}}_{T\_SD\_CD}^{M,aML}$	${\overset{=}{ML}}_{T\_SD\_CD}^{M,SVM}$	${\overset{=}{ML}}_{T\_SD\_CD}^{M,aML}$
Depression	74.01	79.82	74.01	88.60	87.40	92.10
Anxiety	62.20	71.05	72.44	81.58	83.46	85.96
Stress	57.48	68.42	70.07	81.58	85.03	88.59

**Table 10:** Binary accuracy results with and without adding kinematic and statistical features, PCA-mFBCF pipeline for features selection and using autoML.

M	${\tilde{ML}}_{T\_SD\_CD}^{M,aML}$	${\tilde{ML}}_{T\_K\_S\_SD\_CD}^{M,aML}$	${\hat{ML}}_{T\_SD\_CD}^{M,aML}$	${\hat{ML}}_{T\_K\_S\_SD\_CD}^{M,aML}$	${\overset{=}{ML}}_{T\_SD\_CD}^{M,aML}$	${\overset{=}{ML}}_{T\_K\_S\_SD\_CD}^{M,aML}$
Depression	79.82	81.57	88.60	92.98	92.10	100.00
Anxiety	71.05	75.43	81.58	88.60	85.96	100.00
Stress	68.42	71.92	81.58	89.47	88.59	100.00

**Table 11:** Trinary accuracy results for PCA, mFCBF and PCA–mFCBF pipeline for the FS methods and by using AutoML.

M	$ML_{T\_K\_S\_SD\_CD}^{M,aML}$	${\tilde{ML}}_{T\_K\_S\_SD\_CD}^{M,aML}$	${\hat{ML}}_{T\_K\_S\_SD\_CD}^{M,aML}$	${\overset{=}{ML}}_{T\_K\_S\_SD\_CD}^{M,aML}$
Depression	74.56	77.19	81.57	82.45
Anxiety	50.87	57.89	71.92	72.80
Stress	47.36	54.38	65.78	74.56

## Data Availability

Available on request.
